# Tools to Support Self-Care Monitoring at Home: Perspectives of Patients with Heart Failure

**DOI:** 10.3390/ijerph17238916

**Published:** 2020-11-30

**Authors:** Ina Thon Aamodt, Anna Strömberg, Ragnhild Hellesø, Tiny Jaarsma, Irene Lie

**Affiliations:** 1 Centre for Patient-Centered Heart and Lung Research, Department of Cardiothoracic Surgery, Oslo University Hospital, Building 63, Box 4956 Nydalen, 0424 Oslo, Norway; UXIRLI@ous-hf.no; 2 Department of Nursing Science, Institute of Health and Society, University of Oslo, 0318 Oslo, Norway; ragnhild.helleso@medisin.uio.no; 3 Campus Lovisenberg, Lovisenberg Diaconal University College, 0456 Oslo, Norway; 4 Department of Health, Medicine and Caring Sciences, Linköping University, 581 83 Linköping, Sweden; anna.stromberg@liu.se (A.S.); tiny.jaarsma@liu.se (T.J.); 5 Department of Cardiology, Linköping University, 581 83 Linköping, Sweden

**Keywords:** heart failure, self-care, telemedicine, eHealth, mHealth, information and communication technology (ICT)

## Abstract

Self-care monitoring at home can be a challenge for patients with heart failure (HF). Tools that leverage information and communication technology (ICT), comprise medical devices, or have written material may support their efforts at home. The aim of this study was to describe HF patients’ experiences and their prioritization of tools that support, or could support, self-care monitoring at home. A descriptive qualitative design employing semi-structured interviews was used with HF patients living at home and attending an HF outpatient clinic in Norway. We used a deductive analysis approach, using the concept of self-care monitoring with ICT tools, paper-based tools, medical devices, and tools to consult with healthcare professionals (HCPs) as the categorization matrix. Nineteen HF patients with a mean age of 64 years participated. ICT tools are used by individual participants to identify changes in their HF symptoms, but are not available by healthcare services. Paper-based tools, medical devices, and face-to-face consultation with healthcare professionals are traditional tools that are available and used by individual participants. HF patients use traditional and ICT tools to support recognizing, identifying, and responding to HF symptoms at home, suggesting that they could be used if they are available and supplemented by in-person consultation with HCPs.

## 1. Introduction

The home is the primary setting for patients diagnosed with heart failure (HF) to perform self-care monitoring. Self-care monitoring is a decision-making process influenced by reflection where HF patients are encouraged to detect, interpret, and respond to bodily changes that may reflect deterioration of their condition at home [1,2]. However, patients often struggle to distinguish HF-related symptoms and signs indicating a worsening condition from other symptoms related to a chronic HF disease state, comorbidities, or general aging-related changes. It is exactly this difficulty in sorting out these health challenges that can contribute to patients’ delay in seeking care [3]. Furthermore, patients with HF may modify their activities and lifestyle to adapt to their current condition and, as a result, they might not recognize HF-related symptoms and signs indicative of a worsening HF condition [2]. Early symptom identification e.g., unspecified dyspnea and response to symptoms in the elderly living at home with a chronic illness such as HF, can prevent development of a severe condition that requires hospitalization [4].

The European Society of Cardiology’s guideline recommend that HF patients receive regular follow-up by healthcare providers [5]. Symptom monitoring may be supported by family members, informal caregivers, and healthcare professionals in person or through telemonitoring [6]. The traditional approach of in-person follow-up is becoming more and more difficult to accomplish due to shortages of healthcare personnel, and increasing numbers of elderly people with comorbidities [7]. This constraint, together with the focus on promoting shorter hospital stays and at-home follow-up for HF patients, makes it even more challenging for healthcare systems to carry out traditional face-to-face follow-ups. One way to address these issues is to leverage information and communication technologies (ICTs) for the purpose of healthcare systems relief. The use of these technologies is rapidly increasing worldwide to support people’s health and to help them get access to healthcare services [8]. One example of leveraging ICT for use in healthcare is telemonitoring, which employs audio, video, and other telecommunication and electronic information processing technology to monitor a patient’s status at a distance [9]. Different telemonitoring methods have been used that enable patients at home to consult with healthcare professionals, such as non-invasive telemonitoring or structured telephone support [10], out-patient video consultations [11], and mobile phone apps to transmit vital signs or to titrate HF patient’s medication [12,13]. The evidence for non-invasive telemonitoring to support HF patient’s self-care is insufficient for the European guidelines’ recommendation [5], and issues still need to be resolved concerning funding, criteria for patients, what role skilled healthcare professionals should play, and how to provide service before implementation in clinical practice [14]. Moreover, HF patients’ acceptance is considered valuable, and HF patients need to be actively involved in successfully implementing telemonitoring programs [15,16]. More research is needed to facilitate self-care through mHealth and consulting HCPs remotely by the use of video [17,18]. Furthermore, future research needs to explore whether patients will find it acceptable to participate in using non-invasive telemonitoring or video consultations [19]. The present study, therefore, aimed to describe the perspectives of HF patients’ experiences in this context with self-care monitoring tools to support individual and consulting behavior and to determine what priority patients place on novel and traditional tools to support self-care monitoring at home.

## 2. Materials and Methods 

### 2.1. Design

This qualitative descriptive study used semi-structured interviews [20]. Our primary focus was to describe the nature of HF patients’ experience with and opinion of possible future interactions with various kinds of self-care monitoring tools at home. 

### 2.2. Setting

Participants living at home were recruited from an HF outpatient clinic at a university hospital in Norway. In Norway, there are two ways patients diagnosed with HF are referred to the HF outpatient clinic: (1) a cardiologist makes the referral after patients are discharged from the hospital, or (2) their local general practitioner at home makes the referral. In Norway’s HF outpatient clinics, specialist nurses are assigned HF patients, who have regular appointments with their respective nurse at 1-, 2-, 3-, or 6-month intervals, aiming at a stable condition with further follow-up by a general practitioner. According to the HF nurses, the average number of visits in total varies from 4–6 for each patient.

### 2.3. Sample and Recruitment 

During their appointment, HF patients were invited by their HF nurse to talk to a study researcher about participating in our study. A purposive sampling strategy was used with efforts to recruit information-rich informants based on the knowledge of the HF nurses at the outpatient clinic [21]. The inclusion criteria were as follows: adults aged 18 years or older, a primary diagnosis of HF, lived in their own home, and were able to speak and read Norwegian. The researcher gave candidates oral and written information about the study. An appointment was made for the interview after written informed consent was obtained. Twenty-two candidate participants were approached and 19 consented to participate.

### 2.4. Data Collection

We interviewed participating patients by appointments. The interviews were conducted in a private room at the HF outpatient clinic or at the participant’s home (4 of the 19). Prior to the interview, the researcher collected basic demographic information, and then asked the participants about their heart failure diagnosis, treatment, previous hospitalization for HF, and experience with ICTs. Thereafter, the interview started with the researcher presenting photographs of different tools to potentially support self-care monitoring at home. Photo elicitation is a way of bridging the gap between the participant’s and researcher’s views, and is suggested to evoke different kinds of information than words or texts [22]. Moreover, it is suggested that photograph eliciting in interviews is a participative approach [23].

The research team developed the interview guide, which consisted of three parts. Firstly, participants were asked to look at each of the eight photos of tools, illustrating different devices or consulting systems to support self-monitoring of HF symptoms at home, and for each photo they were asked: “What do you think about using this specific tool at home?” We asked predetermined probing questions to get a deeper understanding of how the participants perceived each tool and their potential experience with it: “Do you have any experience with using this tool? Could you say something more about how this tool might support you at home?” [24]. Secondly, participants were asked to suggest any other tools that might support them at home for HF symptom monitoring. Third, participants were asked to rate the eight tools on a scale from 1–8, where 1 was “very interesting to me” and 8 was “not of interest to me”. 

**Figure 1:**
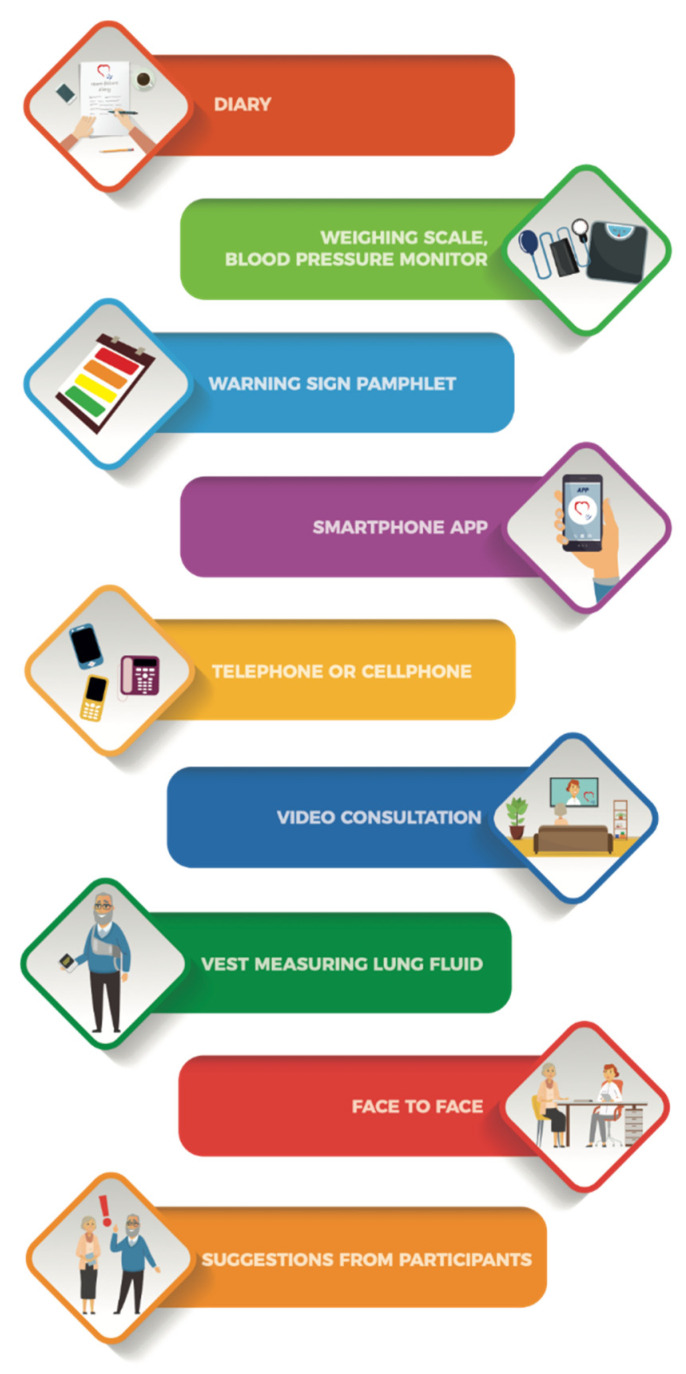
A graphic illustration of tools presented to participants: a diary, weighing scale, blood pressure monitor, warning sign pamphlet, mobile phone application, telephone consultation, lung fluid measuring vest, video consultation, face-to face consultation with HCPs, and suggestions from participants’ of other potential tools to be of support.

The eight tools we selected for this study were based on recommendations of HF guidelines [5] from policymakers in Norway [25] and from reports by the World Health Organization (WHO) [8,26]. The researcher presented photographs of an HF diary used at the outpatient clinic, a body weight scale with a BP and pulse oximeter monitor, and a color-coded pamphlet containing a list of warning signs associated with various HF conditions and instructions (e.g., call primary physician if your shortness of breath increases) [27]. This pamphlet was based on guidelines published on an informational nonprofit website of the European Society of Cardiology designed for heart failure patients [28]. Furthermore, the researcher showed participants photographs of a lung fluid vest (a medical device), and a mobile phone application. Consulting tools, such as a telephone, a video, a face-to-face consultation with an HCP, and suggestions from participants were also presented. A graphic illustration of these tools is presented in Figure 1. 

We conducted three pilot interviews using photographs of potential tools to identify HF symptoms at home with the aim of refining questions and photographs for the interviews. Although these tools were presented to these pilot subjects, they did not rate these tools. The semi-structured interviews were conducted face-to-face from May 2017 to January 2018 and audio recorded; they lasted from 31 min to 61 min. Interviews were conducted by the first author (ITAa). All interviews were conducted in Norwegian, and some participant excerpts were translated into English by the authors for illustrative use in the Results section. 

### 2.5. Deductive Content Analysis

The audiotaped interviews were transcribed verbatim, and transcripts were checked against the audio recordings by two of the authors (ITAa and IL). Deductive content analysis inspired by Elo and Kyngäs [29] was performed with the concept of self-care monitoring [2] and the eight tools as the categorization matrix. The eight tools were divided into four analytical categories to identify HF symptoms as described in the literature and reports. The four analytical categories are: paper-based tools to identify HF symptoms, medical devices to identify HF symptoms, mHealth tools to identify HF symptoms, and tools to consult with HCPs to identify HF symptoms. The authors identified the text data of the participants describing each tool in the transcripts of the interviews. Direct quotations from these interviews are presented in Table 1 and also appear in the narrative of Section 3. 

### 2.6. Rigor

To obtain rigor, the same interviewer conducted all of the interviews in the data collection and was not involved in the participants’ care. Three authors were involved in the decision of coding, analysis, and interpretation, and the decisions were reviewed by two other authors and discussed in the research group until an agreement was reached [29,30]. To ensure credibility, the context and the selection of participants with characteristics and quotes are presented. The authors are experienced nurses, specializing in cardiology, general medicine, and healthcare service, and are established qualitative researchers involved in self-care and telemonitoring research. 

### 2.7. Ethical Considerations

The study was conducted in compliance with the principles of the Declaration of Helsinki [31] and had ethical approval from the Regional Committee for Medical and Health Research in Norway (REC no. 2014/1890). All participants signed a written informed consent form before data collection. Additionally, they all agreed that the researchers could access, read, and collect data from their medical records, and could audiotape their interviews. Two participants became emotionally upset while talking about their illness and self-care; their interviews were paused until they were ready to continue. All participants completed the interview and were encouraged to contact their HF nurse or the researcher after the interview in case they experienced any distress. 

## 3. Results

In total, 19 individuals diagnosed with HF consented to participate. Their mean age was 64 years, ranging from 32–85 years, and twelve lived with a spouse or children and seven lived alone. During the previous 12 months, 12 participants were hospitalized because their HF worsened. Eighteen of the 19 participants had previous experience with various kinds of ICT tools, ranging from 3–50 years of experience. Demographics, HF characteristics, and ICT experience of the included participants are summarized in Table 2. 

Next, we describe the participants’ experience with the presented tools, how the participants perceived the use of each tool in realistic but imaginary scenarios, and their priority of tools to support their self-care monitoring at home. 

### 3.1. Mhealth Tools to Identify HF Symptoms 

#### Smartphone App 

Participants used apps on their smartphones in their everyday life, for example, to read newspapers, to access information about public transportation, or to access their bank account. Some participants used smartphone apps to monitor their activity, sleep, or even to measure blood oxygen saturation, heart rate, blood pressure, and blood glucose levels. Participants stated that an ideal of the future HF app would be to display official information about HF, symptoms and signs of a worsening condition, a list of their medications and a reminder to take their medication at predetermined times. 

Male, 59 years, college, work: “An app with information from healthcare authorities and professionals about HF would be of support for me e.g., sometimes I feel my heart racing and using an application with instructions of what to do as lay down on the sofa, is relevant for me.”

Some participants indicated that an HF app would not be useful for them because they lacked experience using smartphone apps or just had no interest in ICT.

### 3.2. Paper-Based Tools to Identify HF Symptoms

#### 3.2.1. Diary

Several participants used or had previously used a daily diary to support self-care at home. Participants wrote in their diaries, read about HF symptoms and signs, read details about their daily medication intake, and recorded the time of their next doctor or outpatient clinic appointment. They also recorded their weight, blood sugar levels, or blood pressure measurements in their diary. They also expressed a feeling of independence in caring for themselves, independent of a healthcare provider.

Male, 77 years, high school, retired: “I wish to manage the medication myself and not be passive because somebody does it for me. I use this diary for keeping track of my medication.” 

One participant stopped using the diary because he felt that the HF information in the diary made him anxious and worried. 

Male, 68 years, college, retired: “I do not feel good reading about heart failure. I used the diary, but now I have put it away. I feel healthy.” 

#### 3.2.2. Pamphlet with Schematic Warning Signs

Our participants were not familiar with the pamphlet illustrating warning signs. However, they recognized the information in it about HF symptoms and signs, like shortness of breath, that was discussed orally with them by the HF nurse at the outpatient clinic. The participants were able to identify the warning signs in the pamphlet, that were reasons for their hospitalization. Individual participants expressed the need for a pamphlet with HF symptoms, signs, and contact information to support them at home. 

Male, 65 years, college, retired: “This [pamphlet] is relevant to me. I didn’t take the shortness of breath as a warning sign. Looking back, I should have been aware and have taken it more seriously.” 

Other participants pointed out disadvantages of having the pamphlet “in sight”, as it constantly reminded them about their HF illness. 

### 3.3. Medical Devices to Identify HF Symptoms

#### 3.3.1. Weighing Scale

The daily use of a weighing scale at home varied among the participants. The purpose of the weighing scale as a supportive tool was to help participants monitor fluctuations in their body weight, which might signal increased retention of body fluid. They also used the scale to determine whether to use as-needed medication to reduce fluid retention. Not all participants experienced changes in their body weight. Rather, some reported that their weight was stable over a long period of time.

Male, 70 years, high school, retired: “I weigh myself every day and write it in my diary. My weight is stable, varying by a tenth of a decimal point, like 0.1 or 0.2 kg.” 

Some participants indicated that their weight gain was due to eating too much food and not fluid retention. Others expressed their negative associations of using a medical device or presented solutions of removing these devices from their field of vision.

Female, 77 years, university, retired: “I monitor my weight regularly because of my diuretic medication. Lately my weight has gained and I've looked angrily at that weighing scale.”

#### 3.3.2. Vest for Measuring Lung Fluid

One of the earliest signs of HF decompensation is fluid retention [5]. This fluid accumulation can be assessed using a non-invasive lung impedance monitor [32]. Participants were unfamiliar with using a vest as a device for measuring fluid accumulation. They reflected about using such a vest and how it would detect fluid accumulation in the lungs before a severe condition developed. Furthermore, they felt that if it was recommended and assessed by healthcare professionals at the HF outpatient clinic, they believed that it had the potential to be supportive for self-care at home. 

Male, 84 years, university, retired: “All such helpful tools are excellent. I am lucky because I do not have water in my lungs. If you do have water in your lungs, it must be all right to put on the vest and measure [it].”

Some participants reported that detecting fluid accumulation in the legs or abdomen was more important for them and were reasons for being uninterested in the vest.

### 3.4. Tools to Consult With HCP to Identify HF Symptoms

#### 3.4.1. Video Consultation 

None of the participants reported having used video consultation with healthcare professionals. Video consultations were not a possible option at the time of data collection. However, participants used peer-to-peer apps, like Microsoft^®^ Skype, to make video calls with their family, to conduct their work activities, or for educational classes. Participants acknowledged that video consultations would save time, energy, and hospital resources. 

Male, 32 years, university, work: “I can use Skype for those visits that involve only a conversation, without taking any tests or conducting clinical examinations. It saves me time away from work and it must be efficient for the hospital.” 

Some participants were concerned about being part of a future in which digital or video consultations were the only way to receive healthcare, as they viewed them as impersonal. Thus, these participants were not interested in these kinds of tools.

#### 3.4.2. Telephone 

The participants used a phone for various reasons, such as to make emergency calls, to make appointments with their general practitioner, or to obtain their blood test results. Some participants stated that using a telephone was impersonal and not suitable for discussing illness-related topics with healthcare professionals.

Female, 62 years, college, welfare: “I use the telephone to get information about my blood test or to order a taxi. I do not use the phone to consult with healthcare professionals or to ask for advice about my illness.”

Participants suggested that a direct telephone number to reach their healthcare professional would potentially be useful to them, because they tended to spend excessive time on hold before reaching them in their current situation.

#### 3.4.3. Face-to-Face

Having face-to-face meetings with the same HF nurse at the HF outpatient clinic was important to participants to support their self-care at home. They said that being able to see a nurse’s facial expressions in person and to experience non-verbal communication are essential in this type of encounter. The HF nurse’s expertise and knowledge, counseling, manner, educational attainment, 6-min walking test, and supportive personality contributed to the participants’ description of independence at home.

Male, 37 years, high school, work: “I do not always feel that I manage [my self-care] correctly at home … not always, and I find it ok to receive some input [from the nurse]. Once in a while, it is fine to come to the HF nurse [workplace] to confirm that what I am doing at home is correct.” 

One disadvantage expressed by participants regarding face-to-face consultation was the extra time needed to travel to the HF outpatient clinic. 

## 4. Additional Participant Suggestions for Supporting Self-Care at Home 

In addition to rating and discussing the eight tools presented to them at the beginning of the study, the participants underlined the importance of assessing and testing their own body at home to identify and respond to HF symptoms. For example, they would take the stairs to assess whether their shortness of breath at home was stable or worsening. Furthermore, the participants underlined the importance of their spouse or family members to support their self-care monitoring at home by either directly monitoring their physical condition face-to-face or by talking to them on the telephone. 

## 5. Participants’ Prioritizing of the Eight Self-Care Tools 

The top-ranked tool prioritized by participants to support their self-care at home was face-to- face meetings with an HF nurse at the outpatient clinic. Novel and traditional tools e.g., an HF app, video consultation, weighing scale, and diary, were top-ranked by individual participants, whereas telephone consultations and the vest for measuring thoracic fluid accumulation were never ranked as being a priority tool.

## 6. Discussion

This study suggests that individuals living at home diagnosed with HF lack access to reliable ICT tools and remote consultation with HCPs. For tools that were unavailable for our participants, such as HF smartphone apps, video consultation, and a vest to measure excessive lung fluid accumulation, some participants have expectations of support to identify HF symptoms and save their energy, however, only if their familiar HF nurses were involved. Other participants had experience with traditional tools, such as paper-based tools and medical devices, to support identifying changes in HF symptoms at home, with follow-up in a face-to-face consultation with HCPs.

Real-world self-care in HF patients has been enhanced using smartphone apps, an approach that involves healthcare professionals less [33,34]. Our findings of HF patients prioritizing face-to-face consultation is also underlined by Allida et al. (2020), in a systematic review of randomized controlled interventions of mHealth, where HCPs have a role in building confidence in HF patients’ daily life of self-care activities and decision-making [35]. HF patients in previous studies reported that a barrier to engaging in self-care at home is that care is fragmented; that is, consulting with different professionals each time at follow-ups who do not know them or their situation [36]. However, studies show that face-to-face consultation with a nurse has potential clinical benefits, like fewer readmissions and lower mortality [37,38]. Moreover, in a study from the Swedish HF Registry, referral to an HF nurse-led outpatient clinic resulted in improved survival of patients with HF [39]. These findings, as well as those from previous studies with healthcare professionals in HF care, demonstrate that an in-person consultation is a good way of performing patient follow-up in HF care [14]. Engaging HF patients in their own care by using a body weighing scale, a diary, or an HF warning signs pamphlet with contact information is recommended in guidelines for HF self-care [5], and this professional consensus is reflected in our participants’ descriptions and prioritizing of medical devices and paper-based tools. Our participants’ descriptions of an ideal future HF app is one that would detect and alert the user to symptoms and signs of a worsening condition. Although this capacity is lacking in current HF apps, this kind of app has the potential to promote better self-care for HF patients [40] and involves the HF patient’s perspective. Furthermore, having HF patients use telemonitoring or a structured telephone support system from home has an impact on HF patients’ quality of life [41], improves their HF symptoms and signs [41,42], and reduces mortality [42], readmissions, and hospitalization [43]. However, implementing telemonitoring has shown an increase in the use of healthcare resources [44], and findings are not consistent and not part of guideline recommendations [5].

The lockdown due to the COVID-19 pandemic has challenged the follow-up of cardiovascular patients and healthcare systems globally, and has highlighted a need for remote delivering of care, with high income countries’ expert opinion of using technology to support access to care [45] and the urgent need for video consultations [46]. Our participants’ opinions of video consultation is reported in other studies to reduce the burdens carried by HF patients, burdens like spending too much time attending appointments [47] and experiencing distressing symptoms, such as lacking energy [27,41]. Surprisingly, the use of video consultations are reportedly scarce in healthcare services [11,48], despite being popular among patients, clinicians, and policymakers [48]. The WHO advocates the routine use of ICT in future healthcare in Europe [49]. Along the same lines, through their sustainable development goals for health and well-being, the United Nations estimates that by 2030, ICT use in healthcare will be used worldwide [50]. In Norway, policymakers have incentivized the use of outpatient video consultation in ongoing projects by using financial rewards [51]. Our findings of participants lacking available ICT tools contribute to future guidelines and criteria for using video consultation. 

The informants’ suggestions about “listening” to and “testing” their body and eliciting support from others have been reported in previous studies as potential facilitators of self-care [52]. An important finding from the current study for healthcare professionals to heed is that when using self-care tools for home monitoring, our HF patients emphasized that they do not want to read about or be reminded about HF. This may be a way of coping in everyday life when living with HF [53], but it also highlights for healthcare professionals the need to listen to the concerns of their HF patients and individualize their care in person or remotely. 

## 7. Strengths and Limitations

A strength in the present study was that the study materials appeared to be reasonably considered, as certain self-care tools were unfamiliar to the participants, yet they were perceived to be supportive to identify HF symptoms and prioritized by some participants. 

This study has some methodological limitations. First, involving the HF nurses well-known to the HF patients may have contributed to participants engaging in descriptions and prioritizing as a kind of “payment gratitude” to their HF nurses. The variation in the participants’ prioritizing and descriptions indicates that access to support was important, and is a well-known factor that has influence on self-care [54]. We do not believe this is a big limitation, as we did probe for clarification and repeatedly questioned the participants during the interview in order to clearly understand their descriptions of the tools and how they felt about them. A second limitation was that we only interviewed HF patients from one outpatient clinic in Norway, meaning that our study needs to be reproduced with other HF patients living at home (at other HF clinics) to bolster the generalizability of our results. Moreover, using photographs involves the participants [23], and they recognized ICT tools they use in daily life e.g., an application, and responded by asking questions about tools they did not recognize in the photographs. 

## 8. Conclusions

In this study, HF patients reported that their experience with traditional tools to identify changes in HF symptoms varies, and some participants perceive mHealth and novel tools to be of support to identify changes in HF symptoms if HCPs are available for support. When optimizing home self-care programs, our results also suggest that traditional tools in use now and tools currently unavailable are relevant in supporting individual HF patients at home, if familiar healthcare professionals are involved. Finally, with further research, our findings may be useful in guiding the development of remote tools to support self-care monitoring for other kinds of patients with chronic health conditions in Europe and other countries, where in-person consultation is becoming increasingly unmanageable.

## Figures and Tables

**Table 1:** Results of the deductive analysis, including examples of units of analysis under each predetermined tool category in the categorization matrix.

Self-care monitoring	**Category**	**Tool**	**Unit of Analysis**
	Paper-based tools to identify HF symptoms	Diary	Male, 60 years, high school, welfare. “I was hospitalized with swollen legs with wounds and everybody thought I had diabetes. I read in the diary about HF symptoms e.g., swollen legs, shortness of breath. I know the symptoms now and use the diary less”
		Warning signs	Male, 59 years, college, work. “I thought cough sirup would relive my breathing problems, but that did not happen. I ended up in hospital. I might have come to the hospital earlier by using the warning signs. I like the warnings signs, but it’s not available.”
	Medical devices to identify HF symptoms	Weighing scale	Female, 77 years, university, retired. “I manage diuretics on-demand by myself, and I check my weight to see the effect of using and not using diuretics.”
		Vest measuring lung fluid	Male, 60 years, high school, welfare. “We should use technology, but there should be a healthcare professional receiving the information from the [measuring] vest. I am against being my own doctor and seeking answers to my questions online.”
	mHealth to identify HF symptoms	Smartphone App	Female, 62 years, college, welfare. “I do not like too much app technology, but I know how to use it and will use it if needed.”
	Tools to consult with HCPs to identify HF symptoms	Face to face	Male, 59 years, college, welfare. “Nothing can replace to meeting in person. All the other things can only be supplemental. There are a lot of signs that are not possible to transfer by phone such as facial expressions and eye contact.”
		Telephone	Male, 72 years, university, retired. “I am a techno freak; I have experience with HCPs support when I had an alarm. I phoned and we solved it by phone. ”
		Video consultation	Male, 78 years, college, retired. “If I experience shortness of breath, pain or chest pain, I can do it [video consultation]) from my home, using my iPad or computer to talk with the nurse and not having to travel to the hospital.”

Abbreviations: HF, heart failure; HCPs, healthcare professionals; ICT, information and communication technology. Translated from the original Norwegian into English.

**Table 2:** Participant demographics, HF characteristics, and ICT experience (*n* = 19).

Characteristics		*n*
Demographic	Gender	
	Female	3
	Male	16
	Highest education level	
	High school	8
	College/University	11
	Current work status	
	Retired or welfare	15
	Work fulltime	4
Clinical	NYHA class	
	NYHA 1	1
	NYHA 2	11
	NYHA 3	5
	NYHA 4	2
	HF Medications	
	ACEI/ARB	19
	Betablockers	18
	Diuretics	17
	Mineral corticoids	12
	HF Device therapy ^a^	8
	Comorbidities	18
ICT Experience	Experience of using	
	Internet at home	18
	E-mail	18
	E-mail on smartphone	11

Abbreviations: ICT, information and communication technology; NYHA class, New York Heart Association Functional Classification. ACEI, angiotensin converting enzyme inhibitors. ARB, angiotensin receptor blockers. ^a^ Cardiac resynchronization therapy with pacemaker or defibrillator, implantable cardioverter-defibrillator.
